# Uncovering Biological Network Function via Graphlet Degree Signatures

**Published:** 2008-04-14

**Authors:** Tijana Milenkoviæ, Nataša Pržulj

**Affiliations:** Department of Computer Science, University of California, Irvine, CA 92697-3435, U.S.A

## Abstract

**Motivation:**

Proteins are essential macromolecules of life and thus understanding their function is of great importance. The number of functionally unclassified proteins is large even for simple and well studied organisms such as baker’s yeast. Methods for determining protein function have shifted their focus from targeting specific proteins based solely on sequence homology to analyses of the entire proteome based on protein-protein interaction (PPI) networks. Since proteins interact to perform a certain function, analyzing structural properties of PPI networks may provide useful clues about the biological function of individual proteins, protein complexes they participate in, and even larger subcellular machines.

**Results:**

We design a sensitive graph theoretic method for comparing local structures of node neighborhoods that demonstrates that in PPI networks, biological function of a node and its local network structure are closely related. The method summarizes a protein’s local topology in a PPI network into the vector of graphlet degrees called the signature of the protein and computes the signature similarities between all protein pairs. We group topologically similar proteins under this measure in a PPI network and show that these protein groups belong to the same protein complexes, perform the same biological functions, are localized in the same subcellular compartments, and have the same tissue expressions. Moreover, we apply our technique on a proteome-scale network data and infer biological function of yet unclassified proteins demonstrating that our method can provide valuable guidelines for future experimental research such as disease protein prediction.

**Availability:**

Data is available upon request.

## Introduction

The recent technological advances in experimental biology have yielded large amounts of biological network data. One such example is *protein-protein interaction (PPI) networks* (or *graphs*), in which nodes correspond to proteins and undirected edges represent physical interactions between them. Since a protein almost never acts in isolation, but rather interacts with other proteins in order to perform a certain function, PPI networks by definition reflect the interconnected nature of biological processes. Analyses of PPI networks may give valuable insight into biological mechanisms and provide deeper understanding of complex diseases. Defining the relationship between the PPI network topology and biological function and inferring protein function from it is one of the major challenges in the post-genomic era (Schwikowski and Fields, 2000; Hishigaki et al. 2001; Vazquez et al. 2003; Letovsky and Kasif, 2003; Deng et al. 2003, 2004; Brun et al. 2004; Nabieva et al. 2005).

### Background

Various approaches for determining protein function from PPI networks have been proposed. “Neighborhood-oriented” approaches observe the neighborhood of a protein to predict its function by finding the most common function(s) among its neighbors. The “majority rule” approach considers only nodes directly connected to the protein of interest (Schwikowski and Fields, 2000). An improvement is made by also observing indirectly connected level-2 neighbors of a node (Chua et al. 2006). Furthermore, the function with the highest **χ^2^** value amongst the functions of all “*n*-neighboring proteins” is assigned to the protein of interest (Hishigaki et al. 2001). Other approaches use the idea of shared neighbors (Samanta and Liang, 2003) or the network flow-based idea (Nabieva et al. 2005) to determine protein function.

Several global optimization-based function prediction strategies have also been proposed. Any given assignment of functions to the whole set of unclassified proteins in a network is given a score, counting the number of interacting pairs of nodes with no common function; the functional assignment with the lowest score maximizes the presence of the same function among interacting proteins (Vazquez et al. 2003). An approach that reduces the computation requirements of this method has been proposed (Sun et al. 2006).

Cluster-based approaches are exploiting the existence of regions in PPI networks that contain a large number of connections between the constituent proteins. These dense regions are a sign of the common involvement of those proteins in certain biological processes and therefore are feasible candidates for biological complexes. The restricted-neighborhood-search clustering algorithm efficiently partitions a PPI network into clusters identifying known and predicting unknown protein complexes (King et al. 2004). Similarly, highly connected subgraphs are used to identify clusters in networks (Hartuv and Shamir, 2000), defining the relationship between the PPI network size and the number and complexity of the identified clusters, and identifying known protein complexes from these clusters (Pržulj et al. 2004). Moreover, Czekanowski-Dice distance is used for protein function prediction by forming clusters of proteins sharing a high percentage of interactions (Brun et al. 2004).

In addition to protein function prediction, several studies have investigated associations between diseases and PPI network topology. Radivojac et al. (Radivojac et al. 2008) have tried to identify candidate disease genes from a human PPI network by encoding each gene in the network based on the distribution of shortest path lengths to all genes associated with disease or having known functional annotation. Additionally, Jonsson and Bates (Jonsson and Bates, 2006) analyzed network properties of cancer genes and demonstrated greater connectivity and centrality of cancer genes compared to non-cancer genes indicating an increased central role of cancer genes within the interactome.

### Approach

We address the above mentioned challenge as follows. First, we verify that in PPI networks of yeast and human, local network structure and biological function are closely related. We do this by designing a method that clusters together nodes of a PPI network with similar topological surroundings and by demonstrating that it successfully uncovers groups of proteins belonging to the same protein complexes, carrying out the same biological functions, being localized in the same subcellular compartments, and having the same tissue expressions. Since we verify this for PPI networks of a unicellular and a multicellular eukaryotic organism (yeast and human, respectively), we hypothesize that PPI network structure and biological function are related in other eukaryotic organisms as well. Next, since the number of functionally unclassified proteins is large even for simple and well studied organisms such as baker’s yeast *Saccharomyces cerevisiae* (Peña-Castillo and Hughes, 2007), we describe how to apply our technique to predict membership in protein complexes, biological functional groups, and subcellular compartments of yet unclassified yeast proteins. Additionally, we show how the method can be used for identification of potential disease genes.

Our method belongs to the group of clustering-based approaches. However, compared to other methods that define a cluster as a dense interconnected region of a network, our method defines it as a set of nodes with similar topological *signatures* (defined below). Thus, nodes belonging to the same cluster do not need to be connected or belong to the same part of the network.

## Methods

**Figure 1:**
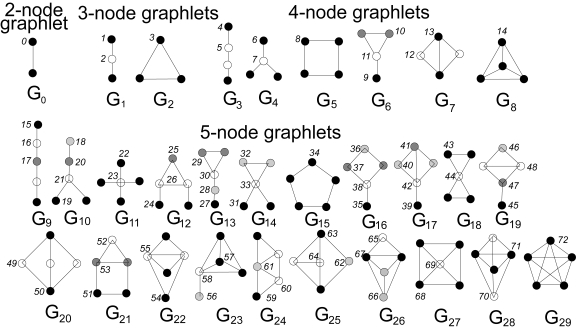
The thirty 2-, 3-, 4-, and 5-node graphlets *G*_0_, *G*_1_, …, *G*_29_ and their automorphism orbits 0, 1, 2, …, 72. In a graphlet G_i_, *i* ∈ {0, 1, …_,_ 29}, nodes belonging to the same orbit are of the same shade (Pržulj, 2006).

Our new measure of node similarity generalizes the degree of a node, which counts the number of edges that the node touches, into the vector of *graphlet degrees*, counting the number of graphlets that the node touches; *graphlets* are small connected non-isomorphic induced subgraphs of a large network (Pržulj et al. 2004) (see Fig. 1). As opposed to *partial* subgraphs (e.g. network *motifs* (Milo et al. 2002)), graphlets must be *induced*, i.e. they must contain all edges between the nodes of the subgraph that are present in the large network. We count the number of graphlets touching a node for all 2–5-node graphlets, denoted by *G*_0_, *G*_1_, …, *G*_29_ in Figure 1; counts involving larger graphlets become computationally infeasible for large networks. Clearly, the degree of a node is the first coordinate in this vector, since an edge (graphlet *G*_0_) is the only 2-node graphlet. We call this vector the *signature* of a node. For example, an outer (black) node in graphlet *G*_9_ touches graphlets *G*_0_, *G*_1_, *G*_3_, and *G*_9_ once, and it touches no other graphlets. It is topologically relevant to distinguish between nodes touching a 3-node linear path (graphlet *G*_1_) at an end, or at the middle node; we provide a mathematical formulation of this phenomenon for all graphlets with 2–5 nodes. This is summarized by *automorphism orbits* (or just *orbits*, for brevity): by taking into account the “symmetries” between nodes of a graphlet, there are 73 different orbits for 2–5-node graphlets, numerated from 0 to 72 in Figure 1 (see (Pržulj, 2006) for details). Thus, the signature vector of a node has 73 coordinates. For example, a node at orbit 15 in graphlet *G*_9_ touches orbits 0, 1, 4, and 15 once, and all other orbits zero times. Thus, its signature will have 1s in the 0th, 1st, 4th, and 15th coordinate, and 0s in the remaining 69 coordinates.

We compute node signature similarities as follows. We define a 73-dimensional vector *W* containing the weights *w_i_* corresponding to orbits *i* ∈ {0, …, 72}. We assign different weights to different orbits for the reasons illustrated below. For example, the differences in orbit 0 (i.e. in the degree) of two nodes will automatically imply the differences in all other orbits for these nodes, since all orbits contain, i.e. “depend on”, orbit 0. Similarly, the differences in orbit 3 (the triangle) of two nodes will automatically imply the differences in all other orbits of the two nodes that contain orbit 3, such as orbits 14 and 72. We generalize this to all orbits. Thus, we need to assign higher weights to “important” orbits, those that are not affected by many other orbits, and lower weights to “less important” orbits, those that depend on many other orbits. By doing so, we remove the redundancy of an orbit contained in other orbits. To compute weights w*_i_*s, each orbit *i* is assigned an integer *o_i_* that is obtained simply by counting the number of orbits that affect orbit *i*. We consider that each orbit affects itself. For example, for orbit 15, *o*_15_ = 4, since it is affected by orbits 0, 1, 4, and itself; similarly, *o*_44_ = 5, since orbit 44 is affected by orbits 0, 2, 3, 11, and itself. We compute *w_i_* as a function of *o_i_* as follows

$$w_{i}=1-\frac{log(o_{i})}{log(73)}.$$

We apply a logarithm function to *o_i_*s to assign higher weights *w_i_*s to the more “important” orbits (those that are not affected by many other orbits). Also, since the maximum value that an *o_i_* can take is 73 (for 2–5-node graphlets), we divide *log*(*o_i_*) by *log*(73) to scale it to [0, 1]. Since an orbit dependency count *o_i_* of 1 indicates that no other orbits affect orbit *i* (i.e. this orbit is of the highest importance), we invert this scaled value of orbit dependencies to assign the highest weight *w_i_* of 1 to orbit *i* with *o_i_* = 1. Clearly, *w_i_* ∈ [0, 1] for all *i* ∈ {0, …, 72} and the formula correctly assigns lower weights to less important orbits.

For a node *u*, we denote by u*_i_* the *i*th coordinate of its signature vector, i.e. *u_i_* is the number of times node *u* touches orbit *i*. We define the distance *D_i_*(*u*, *v*) between the *i*th orbits of nodes *u* and *v* as:

$$D_{i}(u,v)=w_{i}\times \frac{|log(u_{i}+1)-log(v_{i}+1)|}{log(max\{u_{i},v_{i}\}+2)}.$$

We use *log* in the numerator because the *i*th coordinates of signature vectors of two nodes can differ by several orders of magnitude and the distance measure should not be entirely dominated by these large values. Also, by using these logarithms, we take into account the relative difference between *u_i_* and *v_i_* instead of the absolute difference. We add 1 to *u_i_* and *v_i_* in the numerator of the formula for *D_i_*(*u*, *v*) to prevent the logarithm function to go to infinity. We scale *D_i_* to be in (0, 1) by dividing with the value of the denominator in the formula for *D_i_*(*u*, *v*). We add 2 in the denominator of the formula for *D_i_*(*u*, *v*) to prevent it from being infinite or 0. We define the total distance *D*(*u*, *v*) between nodes *u* and *v* as:

$$D(u,v)=\frac{\sum_{\text{i}=0}^{72}D_{i}}{\sum_{i=0}^{72}w_{i}}.$$

Clearly, the distance *D*(*u*, *v*) is in [0, 1), where distance 0 means the identity of signatures of nodes *u* and *v*. Finally, the *signature similarity*, *S*(*u*, *v*), between nodes *u* and *v* is:

S(u,v)=1-D(u,v).

For example, the two outer (black) nodes at orbit 15 in graphlet *G*_9_ have the same signatures, and thus, their total distance is 0 and their signature similarity is 1.

**Figure 2:**
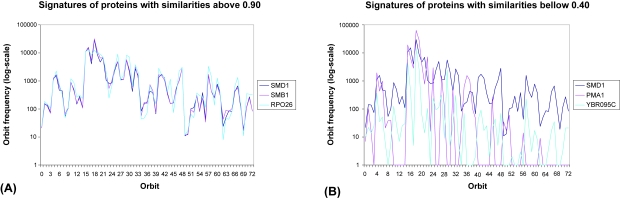
Signature vectors of proteins with signature similarities: (**A**) above 0.90; and (**B**) below 0.40. The 73 orbits are presented on the abscissa and the numbers of times that nodes touch a particular orbit are presented on the ordinate in log scale. In the interest of the aesthetics of the plot, we added 1 to all orbit frequencies to avoid the log-function to go to infinity in the case of orbit frequencies of 0.

We form clusters in a PPI network as follows. For a node of interest, we construct a cluster containing that node and all nodes in a network that are similar to it; we repeat this for each node in the PPI network. According to the signature similarity metric, nodes *u* and *v* will be in the same cluster if their signature similarity *S*(*u*, *v*) is above a chosen threshold. We choose an experimentally determined thresholds of 0.9–0.95. For thresholds above these values, only a few small clusters are obtained, especially for smaller PPI networks, indicating too high stringency in signature similarities. For thresholds bellow 0.9, the clusters are very large, especially for larger PPI networks, indicating a loss of signature similarity. To illustrate signature similarities and our choices of signature similarity thresholds, in Figure 2 we present the signature vectors of yeast proteins in the PPI network of (Krogan et al. 2006) with signature similarities above 0.90 (Fig. 2A) and below 0.40 (Fig. 2B). Signature vectors of proteins with high signature similarities follow the same pattern, while those of proteins with low signature similarities have very different patterns.

## Results and Discussion

### Results

We apply our method to six *S. cerevisiae* PPI networks and three *human* PPI networks. The *S. cerevisiae* PPI networks are henceforth denoted by “vonMering-core” (von Mering et al. 2002), “vonMering” (von Mering et al. 2002), “Krogan” (Krogan et al. 2006), “DIP-core” (Deane et al. 2002), “DIP” (Xenarios et al. 2002), and “MIPS” (Mewes et al. 2002). “vonMering-core” contains only high-confidence interactions described by von Mering et al. (von Mering et al. 2002); it contains 2,455 interactions amongst 988 proteins obtained mainly by tandem affinity purification (TAP) (Rigaut et al. 1999; Gavin et al. 2002) and High-Throughput Mass Spectromic Protein Complex Identification (HMS-PCI) (Ho et al. 2002). “vonMering” is the PPI network containing the top 11,000 high-, medium-, and low-confidence interactions amongst 2,401 proteins described by von Mering et al. (von Mering et al. 2002); the dominant techniques used to identify PPIs in this network are TAP, HMS-PCI, gene neighborhood, and yeast-two-hybrid (Y2H). “Krogan” is the “core” PPI data set containing 7,123 interactions amongst 2,708 proteins obtained by TAP experiments as described by Krogan et al. (Krogan et al. 2006). “DIP-core” is the more reliable subset of the yeast PPI network from DIP (Xenarios et al. 2002) as described by Deane et al. (Deane et al. 2002); it contains 5,174 interactions amongst 2,210 proteins. “DIP” and “MIPS” are the yeast PPI networks downloaded in November 2007 from DIP (Xenarios et al. 2002) and MIPS (Mewes et al. 2002) databases, respectively; they contain 17,201 and 12,525 interactions amongst 4,932 and 4,786 proteins, respectively. The three human PPI networks that we analyze are henceforth denoted by “BIOGRID” (Stark et al. 2006), “HPRD” (Peri et al. 2004), and “Rual” (Rual et al. 2005). “BIOGRID” and “HPRD” are the human PPI networks downloaded in November 2007 from “BIOGRID” (Stark et al. 2006) and “HPRD” (Peri et al. 2004) databases, respectively; they contain 23,555 and 34,119 interactions amongst 7,941 and 9,182 proteins, respectively. “Rual” is the human PPI network containing 3,463 interactions amongst 1,873 proteins, as described by Rual et al. (Rual et al. 2005). We removed all self-loops and multiple edges from each of the PPI networks that we analyzed.

The entire PPI network is taken into account when computing signature similarities between pairs of nodes (i.e. proteins) and forming clusters (see Methods). However, here we only report the results of analyzing proteins involved in more than four interactions. We discard poorly connected proteins from our clusters because they are more likely to be involved in noisy interactions. Similar was done by Brun et al. (Brun et al. 2004). Also, we discard very small clusters containing less than three proteins. For the remaining clusters, we search for common *protein properties*: in yeast PPI networks, we look for the common protein complexes, functional groups, and subcellular localizations (described in MIPS (Mewes et al. 2002)) of proteins belonging to the same cluster; in human PPI networks, we look for the common biological processes, cellular components, and tissue expressions (described in HPRD (Peri et al. 2004)) of proteins in the same cluster.

Classification schemes and the data for the three protein properties that we analyzed in yeast PPI networks were downloaded from MIPS database (Mewes et al. 2002) in November 2007. For each of these three classification schemes (corresponding to protein complexes, biological functions, and subcellular localizations), we define two levels of strictness: the *strict* scheme uses the most specific MIPS annotations, and the *flexible* one uses the least specific ones. For example, for a protein complex “category” annotated by *510.190.900* in MIPS, the strict scheme returns *510.190.900*, and the flexible one returns *510*. Classification schemes and the data for the three protein properties that we analyzed in human PPI networks (corresponding to biological processes, cellular components, and tissue expressions) were downloaded from HPRD database (Peri et al. 2004) in November 2007. In order to test if our method clusters together proteins having the same protein properties, we refine our clusters by removing the nodes that are not contained in any of the yeast MIPS protein complex, biological function, or subcellular localization categories, or in any of the human HPRD biological process, cellular component, or tissue expression categories, respectively.

**Figure 3:**
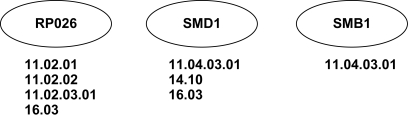
An example of a three-node cluster, consisting of proteins RPO26, SMD1, and SMB1. The categories of biological functions that the proteins belong to are presented bellow the protein names.

In our clusters, we measure the size of the largest common category for a given protein property as the percentage of the cluster size; we refer to it as the *hit-rate*. That is, we compute the hit-rate of a cluster *C* as $Hit(C)=max\frac{N_{p}}{N}$, where *N_p_* is the number of nodes in *C* having a given protein property *p*, and *N* is the total number of nodes in *C*. Clearly, a yeast protein can belong to more than one protein complex, be involved in more than one biological function, or belong to more than one subcellular compartment (and similar holds for human proteins). Thus, it is possible to have an overlap between categories, as well as more than one largest category in a cluster for a given protein property. We illustrate this for biological functions in the cluster presented in Figure 3, consisting of yeast proteins RPO26, SMD1, and SMB1. According to the strict scheme, protein SMD1 is in the common biological function category with protein RPO26 (16.03), as well as with protein SMB1 (11.04.03.01). Thus, there are two largest common biological function categories. The size of the largest common biological function category in the cluster is two and the hit-rate is 2/3 = 67%. For the flexible scheme, all three proteins are in one common biological function category (11) and thus, the size of the largest common biological function category is three and the hit-rate is 3/3 =100%.

We also define the *miss-rate* as the percentage of the nodes in a cluster that are not in any common category with other nodes in the cluster, for a given protein property. That is, we compute the miss-rate of a cluster *C* as $Miss(C)=\frac{U_{p}}{N}$, where *U_P_* is the number of nodes in *C* not sharing any of their protein properties *p* with any other node in *C*, and *N* is the total number of nodes in *C*. For example, in Figure 3, according to the strict scheme, proteins RPO26 and SMB1 are in a common biological function category with SMD1, but they themselves are not in any common biological function category. Although not all three proteins are in the same biological function category and the hit-rate is only 67%, the miss-rate is 0/3 = 0%, since every node is in at least one common biological function category with another node in the cluster. Clearly, the miss-rate for the flexible scheme is also 0/3 = 0%, since the three proteins are in the same biological function category (11) with respect to this scheme. Thus, if a protein belongs to several different categories for a given protein property (which is expected), the hit-rate in the cluster might be lower than 100% (as illustrated in Fig. 3). Therefore, miss-rates are additional indicators of the accuracy of our approach.

**Figure 4:**
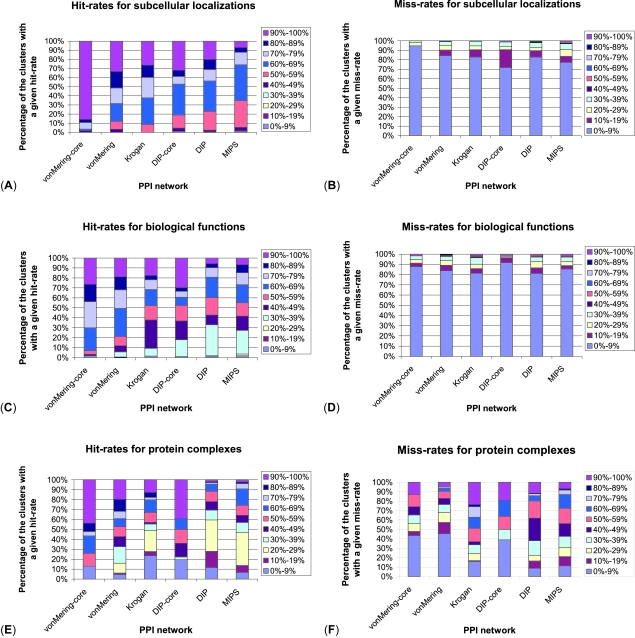
The results of applying our method to the six yeast PPI networks (vonMering-core, vonMering, Krogan, DIP-core, DIP, and MIPS) and the three protein properties (subcellular localizations, biological functions, and protein complexes) in accordance with the flexible scheme: (**A**) hit-rates for subcellular localizations; (**B**) miss-rates for subcellular localizations; (**C**) hit-rates for biological functions; (**D**) miss-rates for biological functions; (**E**) hit-rates for protein complexes; (**F**) miss-rates for protein complexes.

For each of the six yeast PPI networks, the three yeast protein properties, and the two schemes, we measure the number of clusters (out of the total number of clusters in a network) having given hit-and miss-rates. We bin the hit- and miss-rates in increments of 10%. The results for the flexible scheme are presented in Figure 4. For subcellular localizations, in vonMering-core network, 86% of the clusters have hit-rate above 90%; for the remaining five networks, 65% of clusters have hit-rates above 60% (Fig. 4A). For all networks, miss-rates for 72% of clusters are bellow 10% (Fig. 4B). Similarly, for biological functions, the miss-rates in all six networks are under 10% for 81% of the clusters (Fig. 4D). The hit-rates for biological functions are above 60% for 79% of the clusters in both von Mering networks; in the remaining four networks, 57% of the clusters have hit-rates above 50% (Fig. 4C). Finally, for protein complexes, 47% clusters in vonMering-core, vonMering, and DIP-core networks have hit-rates above 60%, 36% of clusters in Krogan and MIPS networks have hit-rates above 50%, and 30% of clusters in DIP network have hit-rates above 40% (Fig. 4E). Miss-rates for protein complexes are bellow 10% for 39% of the clusters in both von Mering networks and in DIP-core network; in the remaining three networks, 33% of the clusters have miss-rates bellow 39% (Fig. 4F).

**Figure 5:**
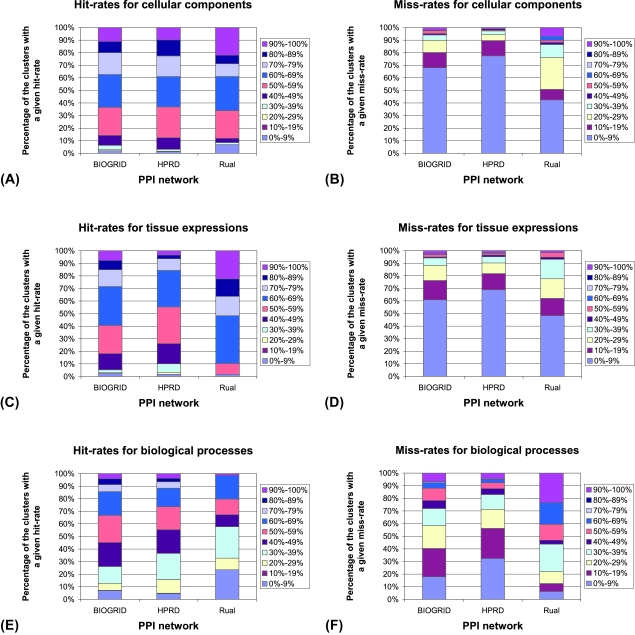
The results of applying our method to the three human PPI networks (BIOGRID, HPRD, and Rual) and the three protein properties (cellular components, tissue expressions, and biological processes): (**A**) hit-rates for cellular components; (**B**) miss-rates for cellular components; (**C**) hit-rates for tissue expressions; (**D**) miss-rates for tissue expressions; (**E**) hit-rates for biological processes; (**F**) miss-rates for biological processes.

Similarly, for each of the three human PPI networks and their three protein properties that we analyzed, we measure the number of clusters (out of the total number of clusters in a network) having given hit- and miss-rates. The results are presented in Figure 5. For cellular components, in all three human PPI networks, 86% of the clusters have hit-rates above 50% (Fig. 5A). Miss-rates for 68% of clusters in BIOGRID and HPRD networks are bellow 10%, while in Rual network 76% of clusters have miss-rates bellow 29% (Fig. 5B). Similarly, for tissue expressions, hit-rates are above 50% for 74% of clusters in BIOGRID and HPRD networks, and for 98% of clusters in Rual network, respectively (Fig. 5C). Miss-rates are lower than 10% for 61% of clusters in BIOGRID and HPRD networks, and for 48% of clusters in Rual network, respectively (Fig. 5D). Finally, for biological processes, hit-rates are above 50% for 55% of clusters in BIOGRID network, for 45% of clusters in HPRD network, and for 33% of clusters in Rual network, respectively. (Fig. 5E). Miss-rates are bellow 29% for 58% of the clusters in BIOGRID network and for 71% of the clusters in HPRD network; in Rual network, 44% of the clusters have miss-rates bellow 39% (Fig. 5F).

To evaluate the effect of noise in PPI networks to the accuracy of our method, we compare the results for the high-confidence vonMering-core network and the lower-confidence vonMering network (Fig. 4). As expected, clusters in the more noisy network have lower hit-rates compared to the high-confidence network. However, low miss-rates are still preserved in clusters of both networks for all three protein properties, indicating the robustness of our method to noise present in PPI networks.

Thus far, we demonstrated that our method identifies groups of nodes in PPI networks having common protein properties. Our technique can also be applied to predict protein properties of yet unclassified proteins by forming a cluster of proteins that are similar to the unclassified protein of interest and assigning it the most common properties of the classified proteins in the cluster. We do this for all 115 functionally unclassified yeast proteins from MIPS that have degrees higher than four in any of the six yeast PPI networks that we analyzed. In Tables 1 and 2, we present the predicted functions for proteins with prediction hit-rates of 50% or higher according to the strict and the flexible scheme, respectively. The full data set with functional prediction hit-rates lower than 50% is available upon request. Note that a yeast protein can belong to more than one yeast PPI network that we analyzed. Thus, biological functions that such proteins perform can be predicted from clusters derived from different yeast PPI networks. We observed an overlap of the predicted protein functions obtained from multiple PPI networks for the same organism, additionally verifying the correctness of our method. Furthermore, there exists overlap between our protein function predictions and those of others (Vazquez et al. 2003).

Finally, we survey the literature and verify that our method successfully predicts biological functions of the following nine proteins from Tables 1 and 2. Our method predicts that protein PWP1 is involved in rRNA processing; this is confirmed by SGD (Cherry et al. 1998) and (Zhang et al. 2004). We also predict that protein IES2 is involved in transcriptional control; this function is verified by (Xu et al. 2007; Svaren et al. 1994; Karnitz et al. 1990). Human OLA1 has been shown to define an ATPase subfamily in the Obg family of GTP-binding proteins (Koller-Eichhorn et al. 2007) indicating that yeast OLA1 might also be involved in protein binding, as predicted by our method. Our method predicts two functions for protein STO1: protein fate (folding), the confirmation of which is indicated by (Grishchuk and McIntosh, 1999), and binding function, the confirmation of which is indicated in SGD (Cherry et al. 1998). Our method correctly predicts that YFR016c is involved in biogenesis of cellular components, since protein encoded by YFR016c interacts with Spa2p that is involved in cytokinesis and cell wall morphogenesis (Shih et al. 2005). It also predicts that YPT35 is involved in cellular transport, transport facilities and transport routes; SGD confirms that YPT35 binds to proteins involved in ER-Golgi or vesicular transport. For protein ILM1, our method predicts DNA repair function; SGD suggests that ILM1 may be involved in mitochondrial DNA maintenance and required for slowed DNA synthesis—induced filamentous growth. We predict that protein YET1 is involved in cellular transport; this function is also indicated in SGD where YET1 is described as an endoplasmic reticulum transmembrane protein and a homolog of human BAP31 protein that is involved in vesicular transport pathways (Wakana et al. 2008). Finally, our method predicts that protein PRM1 is involved in biogenesis of cellular components and SGD suggests that it is involved in membrane fusion during mating.

### Discussion

To our knowledge, this is the first study that relates the PPI network structure to all of the following: protein complexes, biological functions, and subcellular localizations for yeast, and cellular components, tissue expressions, and biological processes for human. Starting with the topology of PPI networks of different organisms that are of different sizes and are originating from a wide spectrum of small-scale and high-throughput PPI detection techniques, our method identifies clusters of nodes sharing common protein properties. Our method accurately uncovers groups of nodes belonging to the same protein complexes in the vonMering-core network: 44% of clusters have 100% hit-rate according to the flexible scheme. This additionally validates our method, since PPIs in this network are obtained mainly by TAP (Rigaut et al. 1999; Gavin et al. 2002) and HMS-PCI (Ho et al. 2002), which are known to favor protein complexes.

Our node similarity measure is highly constraining, since we take into account not only a node’s degree, but also additional 72 “graphletdegrees” (see Methods). Since the number of graphlets on n nodes increases exponentially with *n*, we use 2–5-node graphlets (see Fig. 1). However, our method is easily extendible to include larger graphlets, but this would increase the computational complexity; the complexity is currently *O*(|*V*|^5^) for a graph *G*(*V*, *E*), since we search for graphlets with up to 5 nodes. Nonetheless, since our algorithm is “embarrassingly parallel” (i.e. can easily be distributed over a cluster of machines), extending it to larger graphlets is feasible. In addition to the design of the signature similarity measure as a number in (0, 1], this makes our technique usable for larger networks.

### Future directions

**Figure 6:**
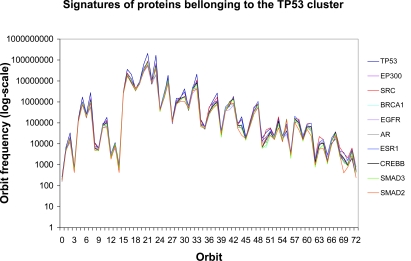
Signature vectors of proteins belonging to the TP53 cluster. The cluster is formed using the threshold of 0.95. The axes have the same meaning as in Figure 2.

Our method can also be applied to disease genes. We consider the set of genes implicated in genetic diseases available from HPRD (Peri et al. 2004). To increase coverage of PPIs, the human PPI network that we analyze is the union of the human PPI networks from HPRD, BIOGRID, and Rual, which consists of 41,755 unique interactions amongst 10,488 different proteins. There are 1,491 disease genes in this PPI network out of which 71 are cancer genes. If graph topology is related to function, then we might expect that genes connected to cancer might have similar graphlet degree signatures. To test this hypothesis, we looked for all proteins with a signature similarity of 0.95 or better when compared to protein TP53. The resulting cluster contains 10 proteins, eight of which are disease genes; six of these eight disease genes are cancer genes (TP53, EP300, SRC, BRCA1, EGFR, and AR). The remaining two proteins in the cluster are SMAD2 and SMAD3 which are members of TGF-beta signaling pathway whose deregulation contributes to the pathogenesis of many diseases including cancer (Gambichler et al. 2007). The striking signature similarity of this 10-node cluster is depicted in Figure 6. To further increase our confidence that local graph topology is related to function, we verified that decreasing the similarity threshold increases the number of nodes in the cluster but decreases the proportion of those nodes that are disease-related. For example, at similarity 0.90, the cluster consists of 39 genes but more than half (21) are non-disease related. Of the 18 disease-related genes, only 8 are cancer genes. In other words, decreasing the threshold from 0.95 to 0.90 barely increases the number of cancer genes but quadruples the total number of matching genes, thus decreasing the specificity by about a factor of 3. A more complete analysis of how topological clustering relates to diseases will be published in a forthcoming paper.

## Conclusions

We present a new graph theoretic method for detecting the relationship between local topology and function in real-world networks. We apply it to proteome-scale PPI networks and demonstrate the link between the topology of a protein’s neighborhood in the network and its membership in protein complexes, functional groups, and subcellular compartments for yeast, and in cellular components, tissue expressions, and biological processes for human. Additionally, we demonstrate that our method can be used to predict biological function of uncharacterized proteins and possibly to identify candidate cancer genes. Thus, this study provides evidence that the graphlet representation of a PPI network has important implications for protein function prediction and gene disease association. Moreover, the method can be applied to different types of biological and other real-world networks, give insight into complex biological mechanisms and provide guidelines for future experimental research.

## Figures and Tables

**Table 1:** Predicted functions with prediction hit-rates of 50% or higher according to the strict scheme for yeast proteins that are unannotated in MIPS and that have degrees higher than four in any of the six yeast PPI networks. The column denoted by “Protein of interest” contains a protein of interest for which the function is predicted. The column denoted by “Degree” contains the degree of a given protein in the corresponding PPI network. The column denoted by “PPI Network” contains the PPI network from which the protein function was derived. The column denoted by “Number of proteins in cluster” contains the total number of proteins in the cluster, including the protein of interest. The column denoted by “Number of unclassified proteins in cluster” contains the number of functionally unclassified proteins in a given cluster, including the protein of interest. The column denoted by “Majority (and predicted) function” contains the common functions amongst at least 50% proteins in the cluster that are also predicted functions for the protein of interest. The column denoted by “Number of proteins in cluster with the majority function” contains the number of nodes in the cluster with the majority function. The column denoted by “Hit-rate” contains the percentage of the total number of proteins in the cluster with the majority function; only the maximum hit-rate is reported for a protein of interest. Finally, the column denoted by “Miss-rate” contains the percentage of annotated nodes in the cluster that do not have a common function with any other annotated node in the cluster.

Protein of interest	Degree	PPI network	Number of proteins in cluster	Number of unclassified proteins in cluster	Majority (and predicted) function	Number of proteins in cluster with the majority function	Hit-rate	Miss-rate
PWP1 (YLR196W)	22	vonMering	23	1	rRNA processing	13	59.09%	13.64%
STO1 (YMR125W2)	42	vonMering	6	1	ATP binding	3	60.00%	20.00%
YMR074C	6	vonMering	3	1	Ribosomal proteins	2	100.00%	0.00%
YMR310C	51	vonMering	7	1	Ribosomal proteins	5	83.33%	0.00%
YNL122C	6	vonMering	3	1	Aminoacyl-tRNA-synthetases	2	100.00%	0.00%
YOR093C	15	vonMering	3	1	Lipid, fatty acid and isoprenoid metabolism	2	100.00%	0.00%
COS6 (YGR295C)	6	DIP-core	9	1	Protein targeting, sorting and translation	4	50.00%	50.00%
YAL027W	19	Krogan	9	3	rRNA processing	3	50.00%	33.33%
YLR455W	19	Krogan	7	2	rRNA processing	3	60.00%	40.00%
PBY1 (YBR094W)	23	MIPS	6	1	Cell wall	3	60.00%	0.00%
YER084W	5	MIPS	7	2	Vacuolar/lysosomal transport	3	60.00%	20.00%
YPT35 (YHR105W)	5	MIPS	7	2	Nuclear transport	3	60.00%	40.00%
ILM1 (YJR118C)	11	MIPS	8	2	DNA repair	3	50.00%	16.67%
					Meiotic recombination	3		
					Protein binding	3		
IES2 (YNL215W)	7	MIPS	5	1	Transcriptional control	2	50.00%	50.00%
YAL018C	9	DIP	3	1	Protein targeting, sorting and translocation	2	100.00%	0.00%
OLA1 (YBR025C)	8	DIP	9	2	Protein binding	4	57.14%	14.29%
COS4 (YFL062W)	22	DIP	5	1	transport facilities	2	50.00%	50.00%
YFR016C	5	DIP	5	1	DNA conformation modification (e.g. chromatin)	2	50.00%	50.00%
YOR220W	6	DIP	5	2	Protein binding	3	100.00%	0.00%

**Table 2:** Predicted functions with prediction hit-rates higher than 50% according to the flexible scheme for yeast proteins that are unannotated in MIPS and that have degrees higher than four in any of the six yeast PPI networks. The columns have the same meaning as in Table 1

Protein of interest	Degree	PPI Network	Number of proteins in cluster	Number of unclassified proteins in cluster	Majority (and predicted) function	Number of proteins in cluster with the majority function	Hit-rate	Miss-rate
PWP1 (YLR196W)	22	vonMering	23	1	Transcription	17	77.27%	0.00%
STO1 (YMR125W2)	42	vonMering	6	1	Protein with binding function or cofactor requirement (structural or catalytic)	5	100.00%	0.00%
					Protein fate (folding, modification, destination)	4		
					Transcription	3		
OLA1 (YBR025C)	10	vonMering	3	1	Cell rescue, defense and virulence	2	100.00%	0.00%
YMR074C	6	vonMering	3	1	Protein synthesis	2	100.00%	0.00%
YMR310C	51	vonMering	7	1	Protein synthesis	5	83.33%	0.00%
YNL122C	6	vonMering	3	1	Protein synthesis	2	100.00%	0.00%
YOR093C	15	vonMering	3	1	Metabolism	2	100.00%	0.00%
COS6 (YGR295C)	6	DIP-core	9	1	Cellular transport, transport facilities and transport routes	6	75.00%	12.50%
					Protein fate (folding, modification, destination)	5		
YAL027W	19	Krogan	9	3	Transcription	4	66.67%	0.00%
					Protein with binding function or cofactor requirement (structural or catalytic)	4		
GDT1 (YBR187W)	6	Krogan	3	1	Transcription	2	100.00%	0.00%
YLR455W	19	Krogan	7	2	Transcription	4	80.00%	0.00%
					Protein with binding function or cofactor requirement (structural or catalytic)	3		
PBY1 (YBR094W)	23	MIPS	6	1	Cell cycle and DNA processing	3	60.00%	0.00%
					Cellular transport, transport facilities and transport routes	3		
					Biogenesis of cellular components	3		
SHU2 (YDR078C)	5	MIPS	4	1	Protein fate (folding, modification, destination)	2	66.67%	0.00%
					Protein with binding function or cofactor requirement (structural or catalytic)	2		
YER084W	5	MIPS	7	2	Cellular transport, transport facilities and transport routes	4	80.00%	20.00%
					Protein fate (folding, modification, destination)	3		
YPT35 (YHR105W)	5	MIPS	7	2	Protein fate (folding, modification, destination)	3	60.00%	0.00%
					Cellular transport, transport facilities and transport routes	3		
EAF6 (YJR082C)	16	MIPS	13	2	Transcription	6	54.55%	9.09%
ILM1 (YJR118C)	11	MIPS	8	2	Cell cycle and DNA processing	4	66.67%	0.00%
YKL061W	7	MIPS	4	1	Metabolism	2	66.67%	33.33%
RAD33 (YML011C)	5	MIPS	9	2	Protein fate (folding, modification, destination)	4	57.14%	14.29%
					Cellular transport, transport facilities and transport routes	4		
IES2 (YNL215W)	7	MIPS	5	1	Cell cycle and DNA processing	3	75.00%	0.00%
SGT2 (YOR007C)	5	MIPS	10	1	Transcription	5	55.56%	0.00%
YPR084W	6	MIPS	4	2	Cellular transport, transport facilities and transport routes	2	100.00%	0.00%
YAL018C	9	DIP	3	1	Protein fate (folding, modification, destination)	2	100.00%	0.00%
					Cellular transport, transport facilities and transport routes	2		
UIP3 (YAR027W)	38	DIP	5	2	Interaction with the environment	2	66.67%	33.33%
YAR028W	11	DIP	3	1	Cellular transport, transport facilities and transport routes	2	100.00%	0.00%
OLA1 (YBR025C)	8	DIP	9	2	Protein with binding function or cofactor requirement (structural or catalytic)	6	85.71%	0.00%
YDL089W	8	DIP	4	2	Protein fate (folding, modification, destination)	2	100.00%	0.00%
YEL068C	5	DIP	6	3	Cell cycle and DNA processing	2	66.67%	0.00%
COS4 (YFL062W)	22	DIP	5	1	Cellular transport, transport facilities and transport routes	3	75.00%	25.00%
					Biogenesis of cellular components	3		
YHR140W	61	DIP	16	2	Metabolism	10	71.43%	7.14%
YET1 (YKL065C)	51	DIP	34	4	Cellular transport, transport facilities and transport routes	16	53.33%	0.00%
YLL023C	22	DIP	17	2	Cellular transport, transport facilities and transport routes	10	66.67%	0.00%
RAD33 (YML011C)	5	DIP	9	3	Metabolism	4	66.67%	0.00%
YNL092W	29	DIP	3	1	Metabolism	2	100.00%	0.00%
PRM1 (YNL279W)	6	DIP	4	1	Metabolism	2	66.67%	33.33%
					Biogenesis of cellular components	2		
YOR164C	6	DIP	6	2	Biogenesis of cellular components	3	75.00%	25.00%
YOR220W	6	DIP	5	2	Protein with binding function or cofactor requirement (structural or catalytic)	3	100.00%	0.00%
					Cellular transport, transport facilities and transport routes	2		
					Interaction with the environment	2		
					Biogenesis of cellular components	2		
